# The mirror neuron system in post-stroke rehabilitation

**DOI:** 10.1186/1755-7682-6-41

**Published:** 2013-10-17

**Authors:** Diana Carvalho, Silmar Teixeira, Marina Lucas, Ti-Fei Yuan, Fernanda Chaves, Caroline Peressutti, Sergio Machado, Juliana Bittencourt, Manuel Menéndez-González, Antonio Egidio Nardi, Bruna Velasques, Mauricio Cagy, Roberto Piedade, Pedro Ribeiro, Oscar Arias-Carrión

**Affiliations:** 1 Physiotherapy Laboratory, Veiga de Almeida University (UVA), Rio de Janeiro, Brazil; 2 Brain Mapping and Sensory Motor Integration, Institute of Psychiatry of Federal, University of Rio de Janeiro (IPUB/UFRJ), Rio de Janeiro, Brazil; 3 NCI, Shanghai, China; 4 Institute of Applied Neuroscience (INA), Rio de Janeiro, Brazil; 5 Laboratory of Panic and Respiration, Institute of Psychiatry, Federal University of Rio de Janeiro (IPUB/UFRJ) - National Institute of Translational Medicine (INCT-TM), Rio de Janeiro, Brazil; 6 Faculty of Medical Sciences, Quiropraxia Program, Central University, Santiago, Chile; 7 Physical Activity Neuroscience, Physical Activity Postgraduate Program, Salgado de Oliveira University (UNIVERSO), Niterói, Brazil; 8 Neurology Unit, Hospital Álvarez-Buylla, Mieres, Spain; 9 Division of Epidemiology and Biostatistic, Institute of Health Community, Federal Fluminense University (UFF), Rio de Janeiro, Brazil; 10 Bioscience Department (EEFD/UFRJ), School of Physical Education, Rio de Janeiro, Brazil; 11 Sleep and Movement Disorders Clinic and Transcranial Magnetic Stimulation Unit, Hospital General Dr Manuel Gea González, Secretaría de Salud, México, DF, México; 12 Sleep and Movement Disorders Clinic and Transcranial Magnetic Stimulation Unit, Hospital General Ajusco Medio, Secretaría de Salud, México, DF, México; 13 Institute of Phylosophy, Federal University of Uberlândia, Uberlândia, Brazil

**Keywords:** Imagery, Imitation, Mirror neurons system, Mirror therapy, Rehabilitation, Stroke

## Abstract

Different treatments for stroke patients have been proposed; among them the mirror therapy and motion imagery lead to functional recovery by providing a cortical reorganization. Up today the basic concepts of the current literature on mirror neurons and the major findings regarding the use of mirror therapy and motor imagery as potential tools to promote reorganization and functional recovery in post-stroke patients. Bibliographic research was conducted based on publications over the past thirteen years written in English in the databases Scielo, Pubmed/MEDLINE, ISI Web of Knowledge. The studies showed how the interaction among vision, proprioception and motor commands promotes the recruitment of mirror neurons, thus providing cortical reorganization and functional recovery of post-stroke patients. We conclude that the experimental advances on Mirror Neurons will bring new rational therapeutic approaches to post-stroke rehabilitation.

## Introduction

Different approaches have been employed to investigate post-stroke rehabilitation [1,2]. It has been shown that the human brain is capable of significant recovery after this type of injury [3,4]. Among its sequels, hemiparesis has been treated with mirror-therapy which promotes cortical changes [5,6]. In particular, sensorimotor disorders in post-stroke patients during the execution or observation of motor action have induced changes to the adjacent cortical penumbra area [7]. Moreover, motion imagination studies have demonstrated efficacies in treating the post-stroke population [8]. Th underlying hypothesis is that “mirror neurons” have been activated during such trainings. These cells were firstly discovered in the premotor cortex of rhesus monkeys by Rizzolatti and colleagues when they analyzed the monkeys observing the researchers’ act of eating up a fruit. These cells were then named because of their property to mirror the observed motor act inside the brain of monkeys [9,10].

Further experiments have verified the existence of mirror neurons in the parietal-frontal circuit, when an animal was exposed to a task of observing a particular action or intention mad by another animal [11,12]. Thus, researchers suggested that mirror neurons are part of a neural system where the observation of an action activates the cortical area of the observer’s brain [10,13-15]. Therefore, the purpose of this review is to describe basic concepts about the current literature on mirror neurons and the major findings regarding the use of mirror therapy and motor imagery as potential tools to promote cortical reorganization and functional recovery in post-stroke patients. The present review is divided in four sections: i) Introduction to Mirror Neuron System: Evidences in Humans; ii) Imitation: The role played by Mirror Neurons; iii) Mirror Neuron System: The Hypothesis of Motor Imagery, and iv) Contributions of the Mirror Neuron System on Post-Stroke Rehabilitation.

### Mirror neuron system: evidence in humans

The mirror neuron system is considered a major breakthrough for neuroscience and it represents one important feature during the evolution of the human brain [16,17]. In this context, several studies analyzed areas where this system participates; in particular, the majority of the experiments in humans and monkeys found mirror neurons in frontal and parietal lobes in tasks involving manual action observation [5,6,18-20]. Moreover, other experiments identified the activation of mirror neurons, specifically in the inferior frontal gyrus and premotor cortex. These findings were replicated in humans during task execution and observation of motor acts with hands, feet and mouth [21-23].

Several tools for cortical stimulation and brain mapping have been employed to uncover the mechanisms behind the activity of mirror neurons. Among them, Transcranial Magnetic Stimulation (TMS) has provided relevant information about the participation of the motor cortex during simple action observation [20,24,25]. Hari *et al*., 1998, investigated the involvement of the mirror neuron system during action observation using magnetoencephalography. With this technique, subjects were instructed to observe stationary or moving stimuli. They observed a suppression of the 15 to 25 Hz activity and concluded that the human primary motor cortex is activated during observation as well as execution of motor tasks. Thus, the mirror neuron system seems to play an important role in human mimicking behavior, and it is activated when an individual observes an action performed by another person. Furthermore, its activation does not depend on memory; i.e., the mirror neuron system is able to identify action complexity, and it unconsciously imitates what we see, hear or perceive [26].

Experiments using electroencephalography (EEG) also demonstrate the existence of mirror neurons in humans during movement observation [27,28]. Cooper *et al*. [28] conducted an EEG study in order to analyze the occurrence of alpha band oscillations over the sensorimotor areas while the participants watched other people yawn. To confirm this hypothesis, researchers showed videos with individuals yawning to the subjects and found that mirror neurons are involved in the recognition of yawning. Additionally, Giromini *et al*. [27], analyzed the EEG μ wave in central areas when subjects watched other people’s movements in different scenarios controlling the amount of external stimuli provided. The results showed that the sensation of motion is capable of triggering activity of mirror neurons even when a small amount of external stimuli is presented. In another study, Oberman *et al*. [29], examined the EEG μ rhythm on the sensorimotor cortex in individuals with autism when compared with controls of the same age [29]. They found that, when children watched the videos with a moving hand or with a bouncing ball or with any visual stimulus, the mirror-neuron system responded dysfunctionally in children with autism compared with controls, as suggested in the “broken mirror” hypothesis. Taken together, mirror neurons have functions that can explain a wide range of human behaviors and neurological disorders [30,31] (See Table 1).

**Table 1:** Clinical studies involving the mirror system

**Study**	**N**	**Tool**	**Procedure**	**Results**	***p *** **value**
Grèzes *et al.*[32]	12	fMRI	Video recordings of objects, grasping pantomimes.	Significant activation in the left intraparietal area during object observation vs baseline.	*p* = 0.001
Montgomery *et al.*[33]	14	fMRI	Videos of communicative hand gestures, object-directed hand movements and word stimuli.	Activations in the inferior parietal lobe and frontal operculum.	*p* < 0.001
Hamilton and Grafton. [34]	20	fMRI	Handed participants watched twelve sets of videos presented in a pseudorandom order and pressed a key if the film was froze in the middle of the action.	A stronger response was found in regions throughout the fronto-parietal circuits, right inferior parietal lobule and right inferior frontal gyrus extending to the inferior frontal sulcus.	*p* < 0.001
Gazzola *et al.*[11]	16	fMRI	Subjects watched either a human or a robot performing various actions. All visual stimuli were video clips lasting between 2.5 and 4 s.	During motor execution active areas were: motor primary in the frontal lobe; sensitive primary and secondary the parietal lobe and the middle temporal gyrus in the temporal lobe.	*p* < 0.001
Michielsen *et al.*[2,23]	22	fMRI	Movement of the hands with observation of the mirror reflex.	The active regions were the precuneus and the posterior cingulate cortex.	*p* < 0.005
Tanaka and Inui. [35]	12	fMRI	Subjects were instructed to imitate presented postures using their right hand or fingers.	Significant activation was observed in Broca’s area.	*p* < 0.001
Heiser *et al.*[36]	14	TMS	Patients watched different videos showing a hand pressing a sequence of 2 (out of 4 possible keys) on a key-press box.	There was a selective deficit of the imitation task for rTMS over the left and right pars opercularis of the inferior frontal gyrus, compared to rTMS over the occipital cortex.	*p* < 0.005
Stefan *et al.*[16]	20	TMS	Task that encoded an elementary motor memory.	Observation of movements led to the formation of a lasting specific memory trace in movement representations that resembled that elicited by physical training.	*p* < 0.005
Oberman *et al.*[37]	11	EEG	Subjects opened and closed their right hand while watching a video of a moving hand.	Consistent pattern of suppression in the frequency band of interest.	*p* = 0.001

*Abbreviations*: *TMS*, Transcranial magnetic stimulation, *EEG*, electroencephalography; μ, mu rhythm, *fMRI*, Functional magnetic resonance imaging, *FMS*, Fugl-Meyer scale, *MP*, Mental practice.

### Imitation and action learning: the role played by mirror neurons

Mirror neurons have been associated with various forms of human behaviors: imitation, mind theory, new skill learning and intention reading [9,38-40]. Studies suggested that humans have a mechanism for copying mental notes of different behaviors, which partly explains how we learn to smile, talk, walk, dance or play tennis. This means that we mentally rehearse or imitate every action observed, whether a somersault or [40,41] a subtle smile, indicating that these cells are used to learn everything from the first basic steps to more graceful accurate movements. Therefore, imitation is involved in learning through the transformation of visual inputs encoded into action by the observer [18].

**Figure 1:**
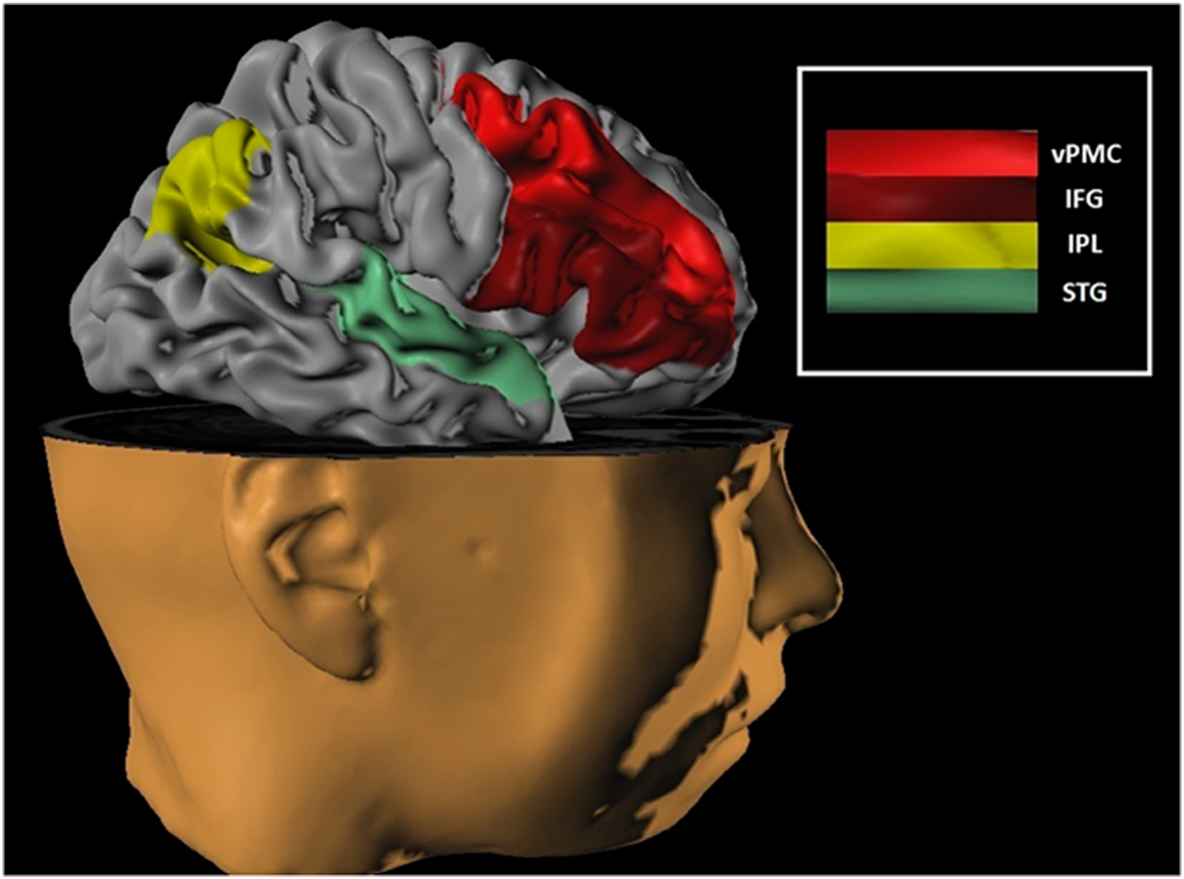
**Neural circuitry for imitation represented in the right hemisphere.** vPMC = ventral premotor cortex; IFG = inferior frontal gyrus; IPL = inferior parietal lobe; STG = superior temporal gyrus. According to the evidence cited in this review, these regions are important to understand the relationship between the mirror neuron areas and the possible therapies in post-stroke patients.

Studies hypothesize that mirror neurons provide a fundamental neural basis for building imitative skills. To clearly define imitative learning, it is necessary to establish three strict criteria, i.e. the emulated behavior must: i) be new to the imitator, ii) reproduce the behavioral strategies of the model, and iii) share the same ultimate goal. Therefore, behaviors that do not meet these criteria should not be regarded as true and imitative and can be explained by other mechanisms such as stimulus enhancement of emulation or “response to facilitation” [42]. Thus, to highlight the imitative process in humans, functional magnetic resonance imaging (fMRI) studies were conducted, instructing the volunteers to observe and imitate a finger movement (imitation task), and to later repeat the same movement in response to space stimuli (task observation/execution). In other experiments the same participants were asked to observe identical stimuli, though without responding to them (the observation tasks). The results showed that cortical activation in imitation tasks is significantly higher than in non-imitative ones [43,44]. Moreover, other fMRI studies have shown that during a simple imitation task, the activation of neuron cells occurs in Brodmann’s area 44, as well as in the parietal cortex. This result supported other experiments showing that the mirror neuron system is involved in human imitation [45,46]. The mirror neurons are present particularly in different cortical areas: inferior frontal, inferior parietal, premotor and occipital cortex [20]. For better visualization (see Figure 1).

In this context, evidences showed that the mirror neuron system is involved in imitation as a response to the observed motor act [35]. Further studies applying TMS found that the mirror neuron system plays a key role in imitation. The use of TMS caused a temporary depression in the caudal region of the left frontal gyrus when the volunteers pressed the keyboard as a response to a red light indicating which key should be pressed. The findings suggested that during imitation, segmentation of cortical action to be imitated occurred and organization of these movements occurred as well [36].

### Mirror neuron system: the hypothesis of motor imagery

Mental practice (MP), or motor imagery, is the internal reproduction of a given motor act, which is repeated several times in order to promote learning or just to improve a given motor skill [8]. Thus, MP results from the conscious access to the intention of moving, and it establishes a relationship between motor events and cognitive perceptions, specifically in post-stroke patients [47,48]. MP can be used according to two different principles: the first consists of internal images, where the individual will perform a mental simulation, and the second applies external image, i.e. the individual watches the movement performed by another individual or by segments of his own body, and this plays an important role in the acquisition of new motor skills [49], improving post-stroke patients rehabilitation. Verma *et al*. [50] evaluated the effectiveness of circuit training with MP in post-stroke individuals, and observed that the gait significantly improved with this practice, i.e. the spasticity was attenuated and ambulation was improved [50]. De Vries *et al*. [47] surveyed 12 subjects who underwent three imagery tasks, in order to examine whether MP improves the recovery of individuals 3 to 6 weeks after the stroke [47]. Results revealed improvements in the ability of visual imagination and suggest that patients with acute stroke, who cannot perform mental practice, should use this modality during the period of rehabilitation. On the other hand, Letswaart *et al*. [51] conducted a cohort study in post-stroke patients (about three months later) with residual weakness in the upper limb [51]. Thirty nine patients underwent four weeks of mental rehearsal of superior movements, 3 times per week, using each arm for 45 minutes. When compared to 32 patients who received normal care, the MP group did not show any significant result, suggesting that motor recovery of post-stroke individuals does not increase with MP. Despite the involvement of mirror neurons in the motor act imitation [35], its activation *via* MP in the early stages of post-stroke rehabilitation was not consistent [51].

### Contributions of the mirror neuron system on post-stroke rehabilitation

The functional damages caused by stroke may be irreversible and compromise the physical functions: cognitive, perceptive, visual and emotional [52]. Thus, physical therapists use many treatments on stroke patients in order to attenuate their sequels. From this point of view, the physical therapy intervention has been implemented based on the neural system. Findings that mirror neurons were activated in monkeys’ cerebral cortex were the first step to research these neurons in human brains [6]. This fact promoted the current treatments of many diseases with the mirror therapy.

The imitation of the movement requires a complex cognitive function that is gradually constructed in several stages including motor observation [53,54]. Considering this, therapies which activate mirror neurons have been used in studies seeking a better post-stroke rehabilitation. In particular, Burns (2008) has shown that motor acts observation in post-stroke patients rehabilitation may accelerate the return to functional activities [55]. Within this context, researchers used fMRI to examine brain activity in post-stroke patients watching a video which contained sequences of mouth, hand and foot movements and they noted that the patients’ cortical areas were activated after observation. A simple exposure to videos showing functional task performances activated the mirror neuron system [7,48]. In particular, the use of mirror therapy has been shown to improve movement recovery, reinforcing motor circuits responsible for the execution of observed actions [8]. This treatment model uses a mirror in such a way that it reflects the action of the healthy limb hiding the affected one. This technique has shown that a neural network responsible for controlling the movement of a particular body region can also be used to control the movements of the contralateral body region. Thus, the idea is to retrain the brain *via* a simple task, where the individual can perform a series of movements with the healthy limb; this is reflected by the mirror and the brain is “tricked” thinking that the movement is performed by the affected limb [1].

The use of mirror therapy in post-stroke patients involves a re-assemblage of the body image in the sensorimotor cortex, which can generate movement limitations, classified as “learned paralysis”. In fact, the fibers that extend from the brain to the spinal cord are deprived of oxygen and suffer an injury, causing a real paralysis. In addition to this, in the early stages of cerebral damage, the penumbra area presents a cellular swelling, temporarily leaving neurons with little or no conduction property. Moreover, during its inactive period, the brain receives only negative visual feedback; this will possibly promote a form of learned paralysis, due to residual mirror neuron functioning. In this case, mirror therapy can potentially reactivate the cortical motor neurons [56]. Therefore, mirror therapy has been used in many clinical instances, because it accelerates the functional recovery of a wide range of sensorimotor disorders, such as post-stroke hemiparesis [57]. Hamzei *et al*. [58] studied the neural plasticity in the primary sensory motor cortex using mirror therapy conducting an experiment in which subjects performed hand movement tasks for 20 minutes every day during 4 days [58]. The authors found that compared to the control group, the performance of the untrained hand improved significantly in the group that used the mirror therapy. Moreover, in the pre-training and post-training analysis, right dorsal and left ventral pre-motor cortex, primary sensorimotor cortex and supplementary motor area were activated. Thus, mirror therapy influences the neural circuitry, which reprograms the motor act by observing the hand trained by the illusory movement of the untrained hand.

In order to examine the clinical effects of mirror therapy and cortical reorganization in 40 post-stroke patients, Michielsen *et al*. [2] divided the subjects into two groups: control and mirror therapy groups; they performed a task using the upper limb one hour per day, five days per week, during six weeks. For such analysis, fMRI and Fugl-Meyer scale (FMS), which evaluates motor function, were used. The FMS results showed that the group with mirror therapy significantly improved its scores, but these changes were not sustained in the follow-up trials. On the other hand, a more balanced activity was observed in the primary motor cortex after fMRI. In another study, Michielsen *et al*. [23] used the mirror therapy in 22 post-stroke patients who performed unimanual and bimanual tasks under two conditions: hand observation (no mirror condition), and observation of the hand reflex in the mirror (mirror condition). They found a significant increase in the posterior cingulate activity and a reduction of 'learned non-use’ (loss of movement ability) patterns during of the movement with the mirror in the bimanual task. They did not find activation during the unimanual condition suggesting that it is not the illusion of a virtual moving hand that causes this activation, but the mismatch between the movement one performs and the movement that is observed [2,23]. Franceschini *et al.*[59] valuated the efficacy of the mirror therapy for upper limb motor impairment in post-stroke patients. In this study, 28 patients with chronic upper limb motor impairment underwent a treatment consisting of watching videos of hand movements for 5 days a week during 4 weeks, and the subjects performed imitation of the movement [59]. Due to the significant findings, they concluded that the observed action can be used as an effective strategy in post-stroke rehabilitations. Thus, mirror neurons can be activated not only using mirror therapy, but also through motor imagery [8].

### MNS and music therapy

Some of the mirror neurons could respond to sounds that are specific for actions, which were named as “audio-visual” mirror neurons [18]. This suggests that combined therapies including both visual and auditory that activate mirror neuron system might be more effective in promoting rehabilitation, which could be, possibly, achieved by online virtual pets [60] or designed multi-media techniques. On the other hand, this suggested that auditory function impairment might be restored with specifically designed MNS training procedures. This idea is yet to be clinically tested.

As a unique and multi-modal stimulus, music transfers visual, auditory, somatosensory and proprioceptive information simultaneously. Interestingly, it has been suggested that music related brain activity involving imitation and synchronization overlapped with MNS brain regions [61,62]. Additionally, the inferior frontal gyrus and the ventral premotor cortex (including Broca’s area) that belong to MNS participated in music execution and listening [63]. For instance, Broca’s area was activated during music perception tasks, active music tasks such as singing, and imagination of playing an instrument [64]. These evidences strongly argued for the potential function of MNS during music-relevant behaviors. It should be noted that in autism patients, individuals with normal (singing) and even superior abilities with specific aspects of music processing (pitch memory and absolute pitch, etc.) could be found as well. This dissociation between singing and speaking is similar to what was observed in Broca’s aphasia patients.

The music based activation of MNS therefore provided alternative option of brain activity manipulation beyond visual training therapy, as well as the possibility of mutli-sensory stimulation. For instance, Melodic intonation therapy (MIT) is one of music-based therapeutic interventions using hand tapping to promote engagement of the sensorimotor network in patients with aphasia [65,66]. These available approaches are ready to be modified from a MNS based perspective for further studies. Music therapy has already been employed to treat some disorders discussed above, such as aphasia [67], stroke [68,69]. It is acknowledged that the psychosomatic effects of music also contributed to the beneficial aspects of music therapy [70,71]; whether MNS activation also mediates such effects are to be examined.

## Conclusion

The mirror neuron system can explain many human behaviors and disorders. Mirror neurons are involved in imitative learning through interactions with neural motor areas in humans. The application of mirror therapy techniques, based on the functions of the mirror neuron system, in post-stroke patients has demonstrated good results, mainly when combined with other therapies. Moreover, the studies showed that the mirror neuron system interacts with vision, proprioception and motor commands, promoting the recruitment of mirror neurons and the cortical reorganization and functional recovery of post-stroke patients. However, many brain areas are not activated in mirror therapy and this factor may compromise the therapy. Also, patients experience fatigue and attention level decrease, which may cause a deficiency in concentration when executing mirror therapy tasks.

## Competing interests

The authors declared that there are no competing interests.

## Authors’ contributions

DC, ST, PR and OAC participated in the definition of the study design and the protocol. Authors DC, ST, PR and OAC managed the literature searches. Authors DC, ST, AEN, PR and OAC wrote the first draft of the manuscript. All authors contributed to and have approved the final manuscript.
